# Mechanical Performance and Failure Modes of High-Strength Adhesives in Aluminum Adherend Joints for Aerospace Applications

**DOI:** 10.3390/ma18194445

**Published:** 2025-09-23

**Authors:** Baojiang Hou, Lifeng Jia, Lisheng Zhang, Bo Xu, Jie Hou

**Affiliations:** 1 School of Aeronautic Science and Engineering, Beihang University, Beijing 100191, China; hennyhobo1001@163.com; 2 State IJR Center of Aerospace Design and Additive Manufacturing, School of Mechanical Engineering, Northwestern Polytechnical University, Xi’an 710072, China; jia_lifeng@mail.nwpu.edu.cn (L.J.); langerouslisa@163.com (L.Z.); xubo0521@mail.nwpu.edu.cn (B.X.)

**Keywords:** adhesive bonding, fracture mechanics, fracture toughness, aluminum adherend joints, TDCB, ENF

## Abstract

Focusing on the practical application requirements of adhesive-bonded structures in aerospace engineering, this study aims to investigate the mechanical performance and failure mechanisms of adhesive interfaces. Adhesive bonding, valued for its uniform load distribution, low stress concentration, superior sealing, and lightweight properties, serves as a critical joining technology in aerospace engineering. However, its reliable application is constrained by complex multimode failure issues, such as cohesive failure, interfacial debonding, and matrix damage. To address these challenges, a comprehensive evaluation of the novel high-strength epoxy adhesive Dq622JD-136 (Adhesive III) was conducted through systematic tests, including bulk tension, butt joint tension, single lap shear, compressive shear, and fracture toughness (TDCB/ENF) tests. These tests characterized its mechanical properties and fracture behavior under mode-I and mode-II loading, with comparative analyses against conventional adhesives HYJ-16 (Adhesive I) and HYJ-29 (Adhesive II). Key findings reveal that Adhesive III exhibits outstanding elastic modulus, significantly outperforming the comparative adhesives. While its normal and shear strengths are slightly lower than Adhesive I, they surpass Adhesive II. A common characteristic across all adhesives is that normal strength exceeds shear strength. In terms of fracture toughness, Adhesive III demonstrates superior mode-II toughness but relatively lower mode-I toughness. These results elucidate the brittle characteristics of such adhesives, mixed failure modes under normal loading, and cohesive failure behavior under shear loading. The innovation of this work lies in systematically correlating the macroscopic performance of adhesives with failure mechanisms through multi-dimensional testing. Its findings provide critical technical support for multiscale performance evaluation and adhesive selection in aerospace joints subjected to extreme thermomechanical loads.

## 1. Introduction

Adhesive bonding features characteristics such as light weight and good sealing performance and has been widely used in aerospace, electronics, and automotive industries [1]. Adhesives can form a uniform bonding layer across extensive contact areas, thereby preventing structural failure caused by localized stress concentration. For parts and structures in aircraft that cannot be connected mechanically, adhesive bonding becomes a more suitable connection method [2,3,4,5].

**Figure 1:**
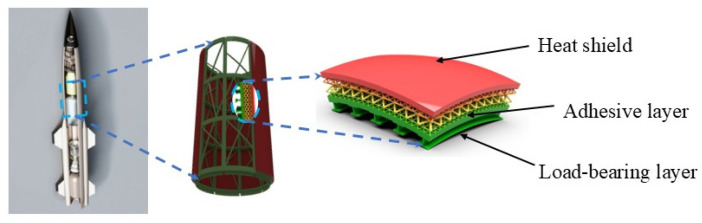
Thermal Protection Structure of the High-speed Aircraft.

For instance, in the multi-layer thermal protection system (TPS) of an aircraft, during high-speed flight, the friction between the aircraft and the air generates high temperatures. The structural shell surface typically includes TPSs, as shown in Figure 1. These TPSs generally consist of a heat-resistant layer, an insulating layer, and a load-bearing layer. Due to the difference in the coefficient of thermal expansion between heat-resistant materials and structural materials, adhesive bonding is required. However, high-speed aircraft needs to endure a series of unique environmental conditions, so the strain capability of the adhesive bonding system must be considered.

To meet these requirements, various modified adhesives have been developed, such as high-strength epoxy adhesives (e.g., DQ622JD-136 adhesive). However, the fundamental mechanical performance parameters (such as the ultimate strength of the adhesive layer, interface fracture toughness, etc.) of newly developed adhesives have not established a systematic quantitative standard.

The constitutive relationship of adhesives exhibits significant nonlinearity, time-dependent characteristics, and strain rate sensitivity, which pose technical challenges for accurate characterization of mechanical properties. Especially under the combined effects of high temperature, high humidity, and salt mist corrosion, adhesives undergo mechanical degradation and aging, coupled with operational conditions, making existing characterization methods unable to accurately predict the actual service performance [6,7]. Furthermore, due to the complex loading conditions in aircraft operating environments, adhesive-bonded structures experience both normal and tangential loads. In multi-layer adhesive joints, the adhesive layer is subjected to both normal and tangential loads. Additionally, the failure modes of adhesive layers are complex; under external loading, adhesives often experience cohesive failure, debonding, and substrate failure. Adhesive joints may experience failure of the adhesive layer or failure at the adhesive interface [8,9].

Many scholars have studied the mechanical properties of adhesive structures. Seong et al. [10] investigated the failure behavior of composite–aluminum single-lap joints, which showed that the mechanical properties of composite–aluminum single-lap joints are lower than those of aluminum–aluminum lap joints. Rudawska [11] compared the lap joint strength of aluminum–composite and titanium–composite and validated the experimental results with numerical simulations. The research indicated that aluminum–aluminum lap joints exhibited higher strength than other lap joints. Ganesh et al. [12] conducted experimental studies on CFRP–metal, CFRP-CFRP, and metal–metal lap joints and analyzed the fracture interfaces. The results showed that metal–metal lap joints had the highest strength, and the failure mode of the lap joints under tensile loading was a mixed mode of cohesive failure and debonding. Golewski et al. [13] investigated the effect of laser surface treatment on aluminum–aluminum adhesive joints, revealing that joints subjected to two-step laser texturing and cleaning treatment exhibited an apparent shear strength that was 20–30% higher than those treated with traditional sandpaper grinding, with a significantly increased proportion of cohesive failure. Additionally, Davide [14] analyzed the load-bearing characteristics of injection-type triple-adhesive joints with different hole diameters, showing that epoxy adhesive injection hole designs with diameters of 12–16 mm enabled the joints to achieve stepwise failure under tensile loading, with overall toughness superior to that of non-porous dual-adhesive systems.

Current research on the mechanical properties of adhesives primarily focuses on the stress analysis of single-lap joints and the influence of adhesive materials themselves on bonding performance, with experimental systems mainly centered on basic tests such as tension, shear, peel, and three-point bending. However, existing studies have obvious limitations: most research related to adhesive layers relies on finite element model simulations or only focuses on either normal or tangential properties in isolation, and systematic experimental studies on adhesives have not been conducted in depth.

To address the limitations of existing adhesive bonding studies, which often rely on finite element simulations or focus solely on isolated normal or tangential properties, this study systematically evaluates the mechanical performance and failure modes of a novel high-strength epoxy adhesive, Dq622JD-136 (Adhesive III), for aerospace TPS. Adhesive III, formulated with a heterocyclic glycidylamine epoxy resin modified by organosilicon compounds and a self-catalytic conjugated amine curing agent, is expected to offer superior mechanical properties compared to conventional adhesives HYJ-16 (Adhesive I) and HYJ-29 (Adhesive II). To guide this evaluation, the following null hypothesis is proposed: H_0_: There are no significant differences in elastic modulus, bulk ultimate tensile strength, mode-I fracture toughness, and mode-II fracture toughness between Adhesive III and Adhesives I and II under room-temperature conditions. This hypothesis tests whether the molecular design of Adhesive III translates to measurable performance advantages. The study employs a comprehensive experimental approach, including bulk tensile tests to determine Young’s modulus, ultimate tensile strength, and failure strain, reflecting the adhesive’s load-bearing capacity. Single lap joint (SLJ) tests under varying temperatures assess the adhesive’s response to thermal fluctuations relevant to aerospace applications. Additionally, tapered double cantilever beam (TDCB) and end-notched flexure (ENF) tests evaluate fracture toughness under mode-I and mode-II loading, respectively, to quantify resistance to crack propagation. These tests enable a detailed comparison of mechanical properties and failure modes among the adhesives, as well as an analysis of normal versus tangential performance within Adhesive III, providing critical data for its application in TPS under extreme thermomechanical loads.

The objective of this study is to systematically evaluate the mechanical performance and failure modes of the novel high-strength epoxy adhesive Dq622JD-136 (Adhesive III) for aerospace TPS applications, delimited to room-temperature testing under mode-I and mode-II loading. By conducting bulk tensile, butt joint tensile, single lap shear, compressive shear, and fracture toughness (TDCB/ENF) tests, and comparing with conventional adhesives HYJ-16 (Adhesive I) and HYJ-29 (Adhesive II), this research provides critical data for adhesive selection in extreme thermomechanical loads. The paper is organized as follows: Section 2 describes the materials used; Section 3 details the experimental methods; Section 4 presents the results and analysis; Section 5 discusses implications; and Section 6 concludes this paper.

## 2. Materials

Adhesive bonding is critical for aerospace thermal protection systems (TPS) due to its ability to provide uniform load distribution, reduced stress concentration, excellent sealing, and lightweight properties. However, conventional low- and medium-temperature curing adhesives, such as epoxy, polyurethane, and silicone-based formulations (curing at ≤120 °C), offer shear strengths of only 1–5 MPa at 200 °C, insufficient for TPS bonding in advanced aerospace structures like re-entry vehicle heat shields or hypersonic aircraft panels. Phenolic adhesives achieve higher shear strength (~10 MPa at 200 °C) but require curing temperatures of 150–180 °C, incompatible with TPS assembly processes. To address these challenges, this study evaluates the mechanical performance and failure modes of a novel high-strength epoxy adhesive, Dq622JD-136 (Adhesive III), designed for TPS bonding in extreme thermomechanical environments.

Adhesive III, formulated with a heterocyclic glycidylamine epoxy resin modified by organosilicon compounds and a conjugated amine-based curing agent, enables self-catalytic curing at 110 °C, offering high strength, low volatility, and minimal shrinkage. These properties make it suitable for TPS applications, such as bonding heat-resistant ceramic tiles to load-bearing aluminum structures in aircraft re-entry modules or high-speed aircraft thermal protection composites, where it withstands high shear loads and thermal cycling in aerodynamic heating environments. Three adhesive formulation series were explored: aldehyde-modified, phenolic-modified, and silicon-modified epoxy matrices, with the silicon-modified series selected for its optimal balance of high-temperature performance and process compatibility. For comparison, two conventional adhesives were tested: HYJ-16 (Adhesive I), an amine-cured epoxy resin used in general aviation structures like wing skin assemblies and generator components, offering robust creep resistance and room-temperature curing for applications in moderate thermal conditions; and HYJ-29 (Adhesive II), a nitrile rubber-modified epoxy matrix with imidazole-based curing agents, commonly applied in automotive composite panels, electronics encapsulation, and high-speed aircraft TPS under severe aerodynamic heating and environmental stress, providing enhanced toughness, heat resistance, and chemical durability. Adhesive III’s molecular design overcomes these limitations, achieving superior stiffness and mode-II fracture toughness, making it a promising candidate for TPS bonding under high shear and thermal loads.

Specimens were prepared at 23 °C and 45% relative humidity to ensure consistency, with manufacturing parameters detailed in Table 1. Adhesive III’s preparation involved weighing the curing agent at a 1:12 mass ratio to the epoxy resin, melting it at 80 °C, mixing with resin and fillers at 300 rpm for 5 min, and resting the mixture for 30 min to remove air bubbles. A four-step curing process was implemented in a programmable oven to achieve full cross-linking and minimize thermal stress: (1) 24 h at 23 °C (±1 °C) with 45% (±5%) humidity to prevent moisture-induced voids; (2) heating to 80 °C at 2 °C/min, held for 2 h; (3) heating to 100 °C at 1 °C/min, held for 6 h; and (4) heating to 110 °C at 1 °C/min, held for 2–4 h based on real-time dielectric sensor monitoring, followed by cooling to 23 °C at 2 °C/min to reduce thermal mismatch stress with aluminum adherends.

## 3. Experimental Methods

### 3.1. Experiment Design

To evaluate the mechanical properties of adhesives for aerospace applications, this study employs a comprehensive experimental framework to assess bulk and interfacial performance under diverse loading conditions. Tests were conducted at 23 °C using a WD4050 electronic universal testing machine (Changchun Kexin Testing Instrument, Changchun, China, 10 kN capacity). The experimental program includes bulk tensile tests to determine Young’s modulus, ultimate tensile strength, and failure strain, reflecting the adhesive’s intrinsic load-bearing capacity. Butt joint tensile tests measure normal bonding strength, while single lap shear and compressive shear tests evaluate tangential strength under ASTM D1002 [15] and D905 [16] standards, respectively. Tapered double cantilever beam (TDCB, ASTM D3433 [17]) and end-notched flexure (ENF) tests quantify mode-I and mode-II fracture toughness, respectively, to assess resistance to crack propagation. High-strength 2A12 aluminum alloy (tensile strength: 470 MPa, elongation at break: 5–12%) was used as the adherend. Surfaces were grit-blasted to Ra2.5 μm and degreased with acetone to ensure consistent interfacial bonding. Adhesives were applied at a uniform 0.2 mm thickness, secured under 0.5 MPa pressure in custom fixtures, and cured following the parameters specified in Table 1.

For TDCB and ENF tests, glass fiber plates maintained consistent adhesive thickness in non-bonded regions. Each test used five specimens, with adhesive layer thickness verified microscopically (0.2 ± 0.02 mm) to ensure uniformity. This systematic approach enables a robust comparison of mechanical properties and failure modes across Adhesive III and conventional adhesives, addressing both normal and tangential performance under conditions relevant to aerospace thermal protection systems.

### 3.2. Specimens and Data Process

For the characterization of bulk tensile properties, dumbbell-shaped specimens were prepared in accordance with ASTM D638 [18]. The adhesives were poured into molds and cured in a forced-air oven under specific temperature-time conditions, after which excess adhesive was removed, and the specimens were demolded.

The cured blocks were precision-machined using a CNC (Computer Numerical Control) milling machine (Shenyang, China) with a diamond cutter, and the thickness was monitored in real time using a digital thickness gauge (Ningbo, China) at 5 random points to ensure the final thickness of 3.5 mm ± 0.05 mm. Specimens with thickness deviation exceeding ±0.05 mm were discarded to avoid affecting tensile stress calculation.

**Figure 2:**
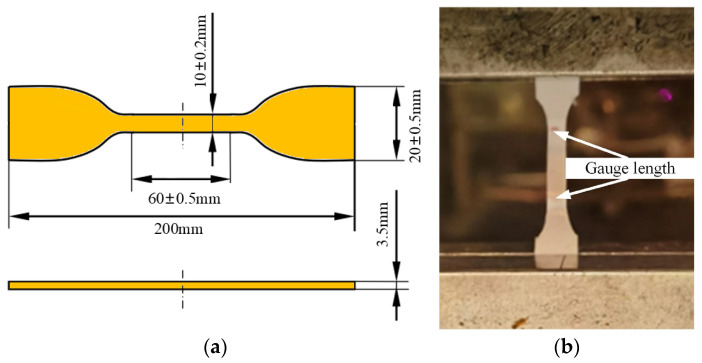
(**a**) Dimension of dumbbell-shaped specimens; (**b**) tensile test set-up.

The cured blocks were precision-machined into 3.5 mm-thick specimens, as shown in Figure 2. The number of test specimens is 5. The quasi-static tensile tests were conducted on a universal testing machine at a constant speed of 10 mm/min.

To calculate the tensile elastic modulus, based on the principle of intercepting the linear segment, strain characteristic points ε_1_ = 0.05% and ε_2_ = 0.25% were selected. The tensile elastic modulus *E* is calculated following:

(1) $$E=\frac{\sigma_{2}-\sigma_{1}}{\varepsilon_{2}-\varepsilon_{1}}$$

and the tensile strength is calculated as:

(2) $$\sigma_{t}=\frac{F_{\max}}{bh}$$

where $\sigma_{t}$ is ultimate tensile strength, and $F_{\max}$ is the maximum tensile load, *b* and *h* is the width and thickness of the specimen.

**Figure 3:**
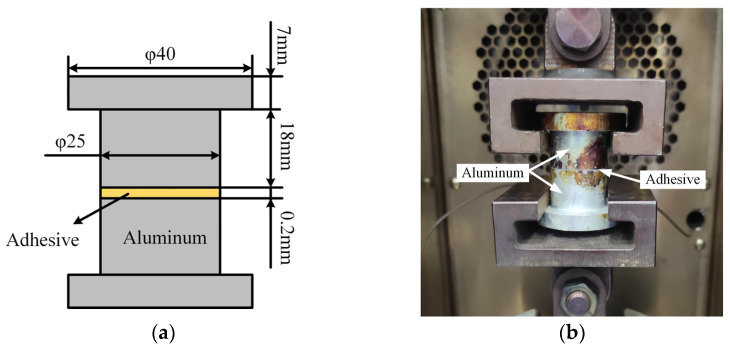
(**a**) Dimension of Adhesive Bonds specimens and; (**b**) butt joint tensile test set-up.

The metal butt joint tensile test obtains the quasi-static tensile strength of adhesive bonds. Orthogonal peel specimens, designed according to ASTM D897 [19], and tension tests are employed. Specimens were prepared with flat surfaces to ensure a uniform adhesive layer at the interface. The surfaces were rigorously cleaned using acetone to eliminate dust and particulate contamination, adhering to the adhesive manufacturer’s specifications. The test specimens and the assembly of specimens mounted on the testing machine are shown in Figure 3. The loading speed of the specimen is 2 mm/min. The number of test specimens is 5. Start the machine and load until the specimen is damaged. Record the maximum load at the time of failure and the type of specimen failure, including cohesive failure, adhesive failure, and metal failure.

The fracture characteristic can be used to quantitatively calculate the tensile strength of adhesive bonds following:

(3) $$\sigma_{x}=\frac{4F_{\max}}{\pi d^{2}}$$

where $\sigma_{x}$ is the tensile strength, $F_{\max}$ is the maximum load during the loading process, and *d* is the radius of the bonding area.

**Figure 4:**
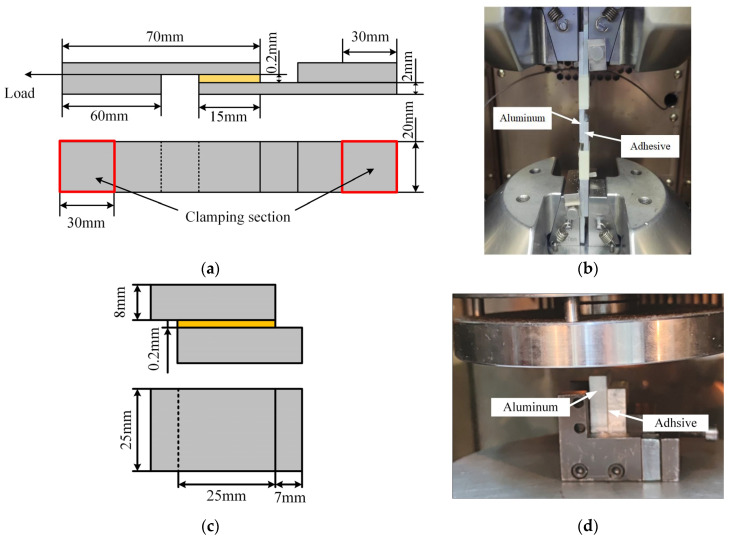
(**a**) Dimension of tensile shear specimens and; (**b**) tensile shear tensile test set-up; (**c**) Dimension of compressive shear specimens; (**d**) compressive shear test set-up.

Single lap shear specimens conforming to ASTM D1002 [15] were prepared to characterize the interfacial shear strength. To further probe into the mechanical behavior of bonded joints under tangential loads, compression shear tests (Figure 4b) were additionally carried out in accordance with ASTM D905 [16]. Surface preparation of adherends consisted of grit blasting and cleaning with acetone. The number of test specimens is 5. Application of the adhesive was done manually, followed by manual positioning of the adherends with application of pressure on the joints. The loading speed of the specimen is 2 mm/min.

The failure mechanisms of both tests, namely interfacial debonding or adhesive shear failure, were analyzed to verify the consistency of the bonding process. In practical applications, adhesive structures frequently endure combined tensile/compressive loads. Thus, the combined test results serve as the foundation for full-load structural design. The tensile/compressive shear strength is calculated as follows:

(4) $$\tau =\frac{F_{\max}}{A}$$

where τ is the strength values shear. $F_{\max}$ is the maximum load during the loading process. *A* is the bonding area of the adhesive test specimen.

**Figure 5:**
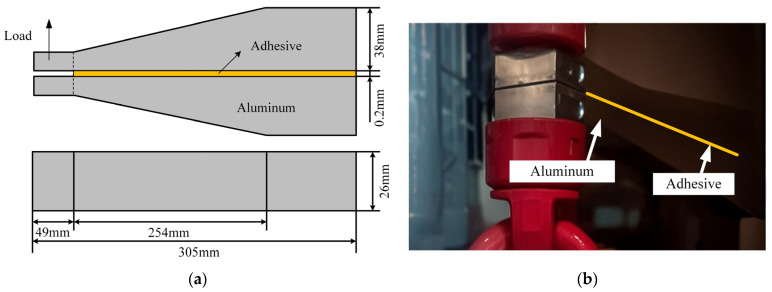
(**a**) Dimension of TDCB specimen and (**b**) TDCB test set-up.

In fracture mechanics, crack propagation modes are defined as two types: Mode I failure mode, also known as the opening-mode failure, where the load is perpendicular to the crack propagation plane; and Mode II failure mode, also referred to as the sliding-mode failure, where the external load is parallel to the crack propagation plane. In this study, experiments were conducted on the two crack propagation modes, respectively. *G*_IC_ is obtained through the TDCB according to ASTM D3433 [17], Method 106. The test specimens and the assembly of specimens mounted on the testing machine are shown in Figure 5. The loading speed of the specimen is 2 mm/min. The number of test specimens is 5. The critical fracture energy *G*_IC_ of this process can be accurately measured as:

(5) $$G_{\mathrm{IC}}=\frac{4P_{c}^{2}}{Eb^{2}}m$$

where *G*_IC_ is the critical fracture energy, *P_c_* is the critical load, *m* is the tapering ratio of the specimen, *E* is the elastic modulus of the specimen matrix material, *b* is the width of the specimen. The tapering ratio *m* needs to be determined through a secondary calculation following:

(6) $$m=\frac{3a^{2}}{H^{3}}+\frac{1}{H}$$

where *a* is the crack length, the distance from the crack tip to the center of the loading pin hole, with the unit of mm; *H* is the actual thickness of the single substrate of the specimen, with the unit of mm.

**Figure 6:**
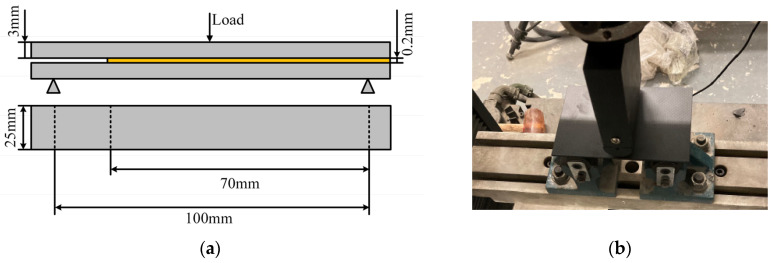
(**a**) Dimension of ENF specimen and (**b**) ENF test set-up.

In accordance with ASTM D7905 [20], the ENF specimen is used to accurately characterize the Mode II slip fracture energy during crack propagation, establishing a quantitative comparison system for normal/tangential energy dissipation. The number of test specimens is 5. The test specimens and the assembly of specimens mounted on the testing machine are shown in Figure 6. The loading speed of the specimen is 2 mm/min. The ENF test is employed to determine the tangential fracture energy *G*_IIC_ of adhesives and investigate their failure modes under tangential loads, with the tangential fracture energy calculated as:

(7) $$G_{\mathrm{IIC}}=\frac{9P^{2}a^{2}}{16EW^{2}H^{3}}$$

where *G*_IIC_ is fracture energy, *P* is the critical load, *E* is the elastic modulus of the adherend, *W* is the width of the specimen, *a* is the effective crack length, and *H* is the actual thickness of a single-piece matrix of the specimen.

To ensure the reliability and repeatability of experimental data, CV was used to quantify the dispersity of key mechanical parameters across 5 replicate specimens for each adhesive. The CV is defined as the ratio of the standard deviation to the mean, with lower CV values indicating higher data consistency. For all tests in this study, the CV of key parameters was consistently <10%. These low CV values confirm the homogeneity of specimen preparation and the stability of the testing system.

## 4. Results

### 4.1. Bulk Tensile Test

**Figure 7:**
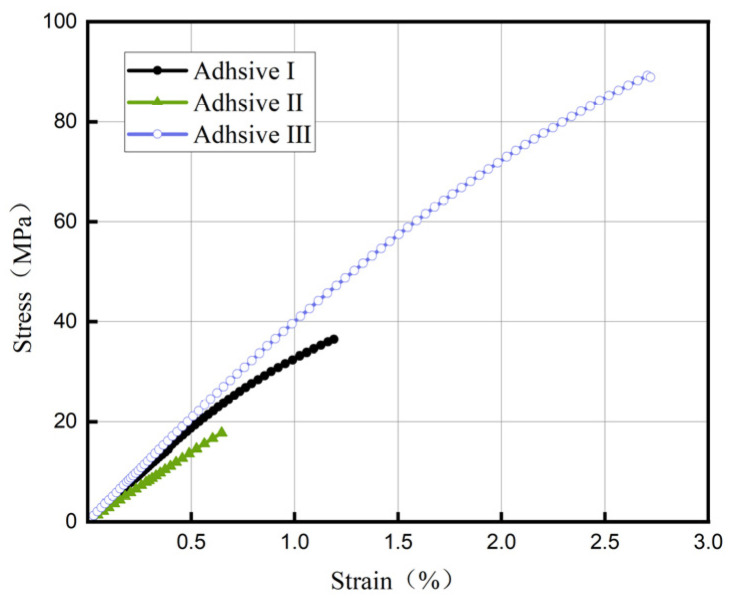
Tensile stress–strain curves of three adhesives.

**Figure 8:**
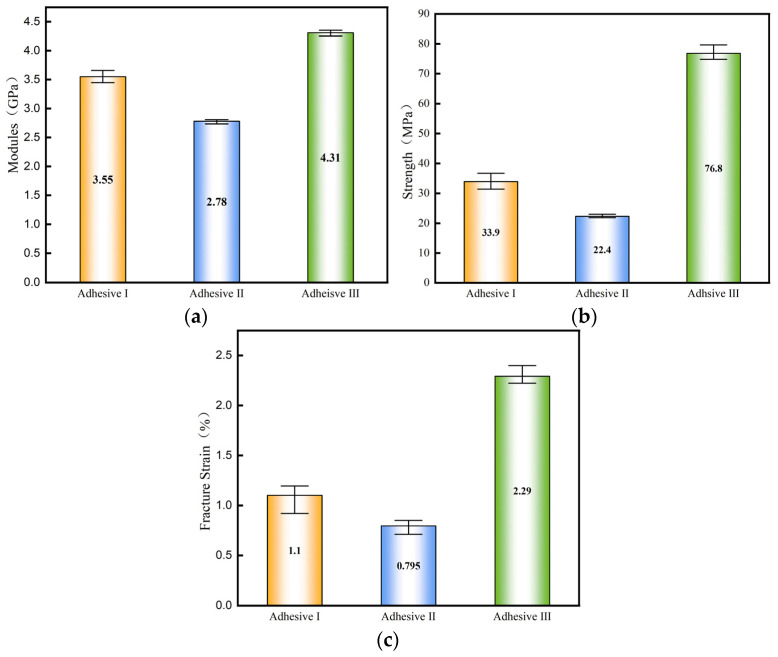
Comparison of parameters in bulk tensile tests of adhesives: (**a**) modulus, (**b**) fracture strength, (**c**) fracture strain.

Figure 7 presents representative stress–strain curves (with strain derived as engineering strain) for the three adhesives. Adhesive III exhibits a steeper curve, indicating a higher elastic modulus of 4.31 GPa, which is 21.4% and 55.0% greater than Adhesive I (3.55 GPa) and Adhesive II (2.78 GPa), respectively. Adhesive III shows no distinct yield point in bulk tensile tests, confirming its brittle behavior. Ultimate tensile strength data in Figure 8 reveal that Adhesive III significantly outperforms the others, surpassing Adhesive I by 126.5% and Adhesive II by 242.9%. Error bars reflect data variability (*n* = 5, CV < 10%), attributed to minor defects in specimen preparation, such as air bubbles or uneven mixing.

### 4.2. Butt Joint Tension Test

**Figure 9:**
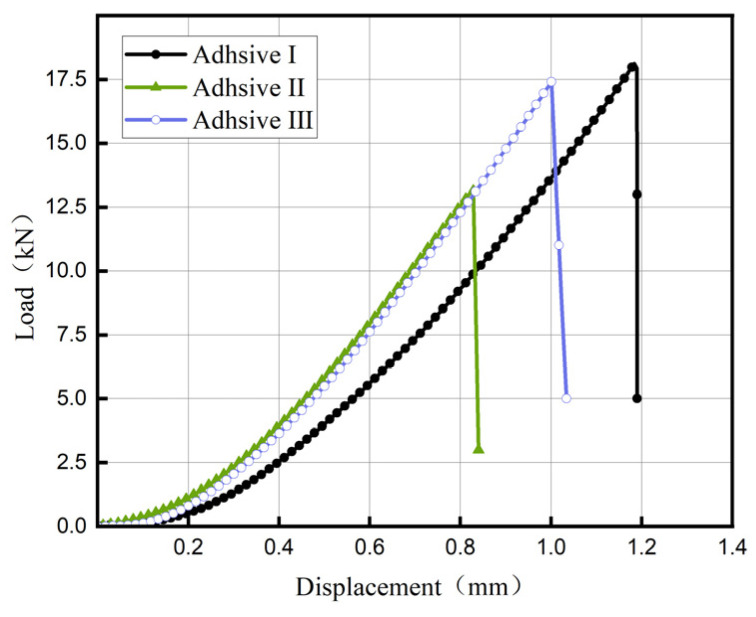
Load–displacement Curve of the Tensile Test for Metal Butt Joints.

**Figure 10:**
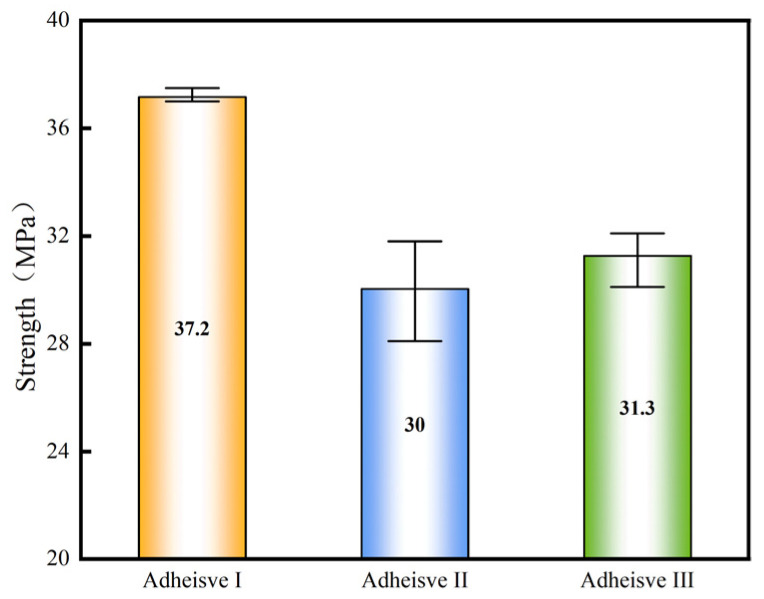
Comparison of the Normal Mechanical Properties of Adhesives.

Figure 9 displays load–displacement curves for the three adhesives. In the initial linear phase, load increases proportionally with displacement; at a critical displacement, the load drops abruptly to zero, indicating brittle fracture of the adhesive layer. Adhesive I exhibits the highest butt joint tensile strength at 37.2 MPa, followed by Adhesive III at 31.3 MPa, slightly higher than Adhesive II at 30.0 MPa (Figure 10, *n* = 5, CV < 7%).

### 4.3. Shear Test

**Figure 11:**
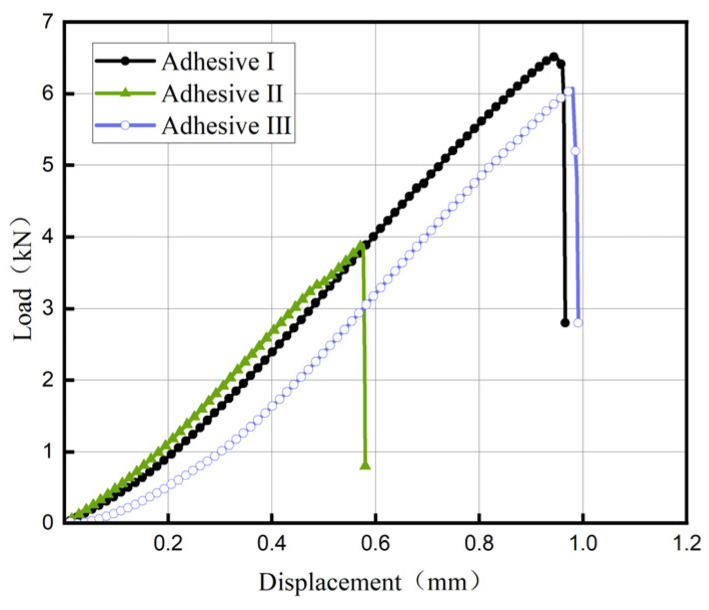
Load–displacement Curve of the Tensile Shear Test.

**Figure 12:**
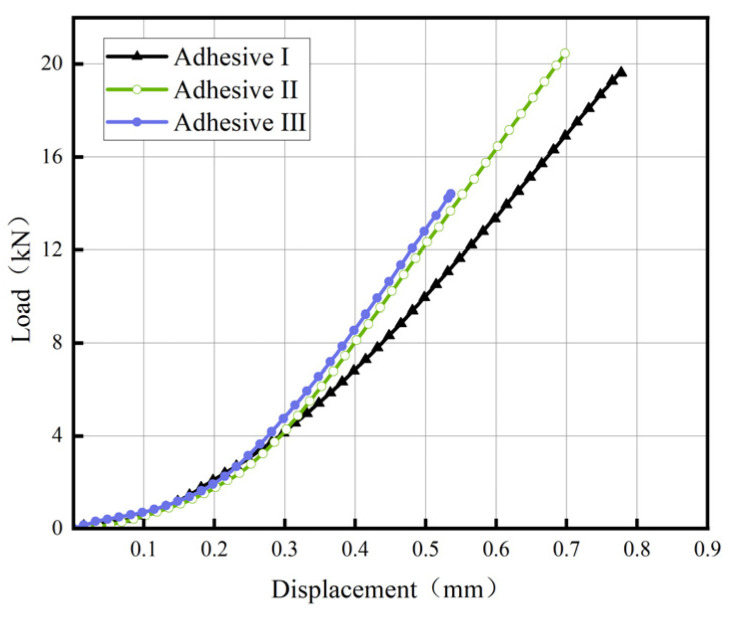
Load–displacement Curve of the compressive Shear Test.

**Figure 13:**
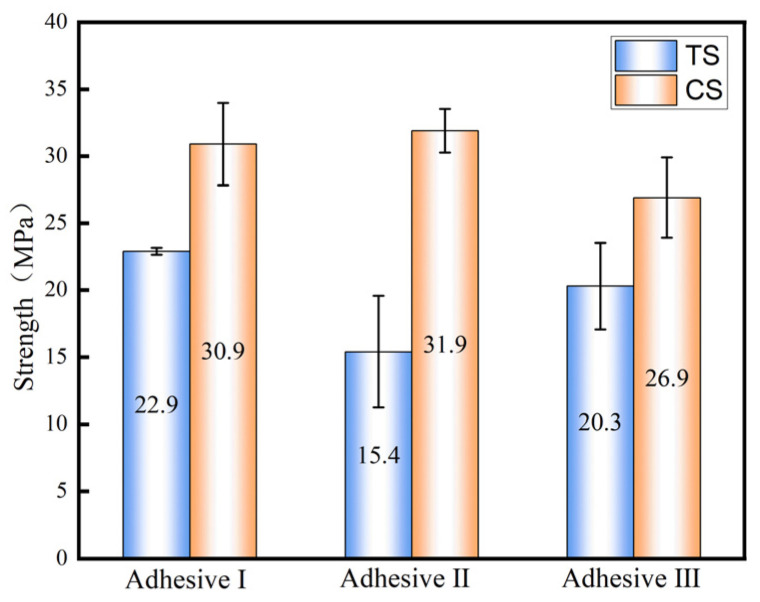
Comparison of strength between compression shear test and tensile shear test for three types of adhesives.

Figure 11 and Figure 12 show load–displacement curves for tensile shear and compressive shear tests, respectively. All three adhesives exhibit brittle behavior under shear loading, with a short damage evolution phase. Figure 13 compares tensile shear (TS) and compressive shear (CS) strengths. Compressive shear strengths consistently exceed tensile shear strengths, likely due to reduced stress concentration during compressive shear loading. Adhesive III’s tensile shear strength is 20.3 MPa, comparable to Adhesive I (22.9 MPa) and 32% higher than Adhesive II (15.4 MPa) (*n* = 5, CV < 9%).

### 4.4. Fracture Tests

**Figure 14:**
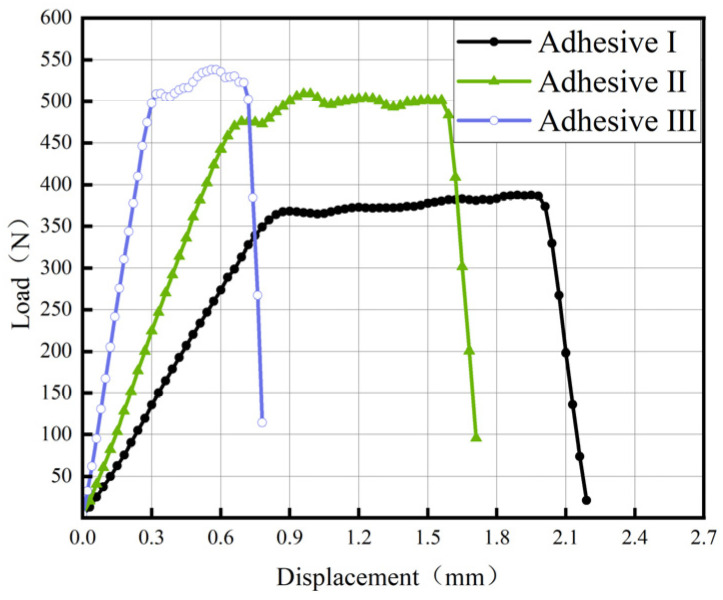
Load–displacement Curve of the TDCB test for Adhesive III.

**Figure 15:**
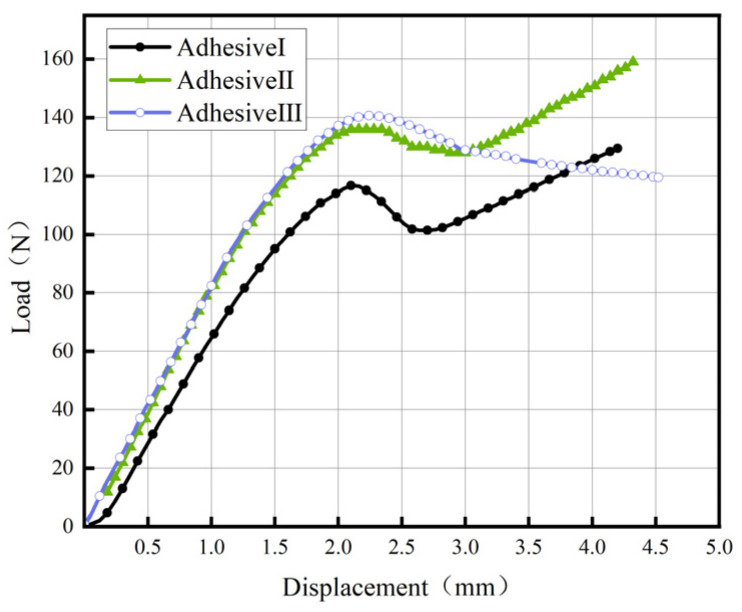
Load–displacement Curve of ENF Test for Adhesive III.

Figure 14 and Figure 15 present representative load–displacement curves for TDCB and ENF of three adhesives. In TDCB tests, load increases linearly with displacement until crack propagation begins. Due to the tapered geometry, the energy release rate remains constant during crack propagation, manifested as a plateau in the curve. In ENF tests, the load peaks and then decreases as the crack propagates; subsequent load increases are not used for calculating mode-II fracture energy (*G*_IIC_). All fracture surfaces exhibit cohesive failure, with no observed interfacial debonding.

Table 2 details the fracture toughness data of the adhesives, revealing a distinct performance trade—off for Adhesive III. Its mode—I fracture toughness (*G*_IC_ = 0.0742 N/mm) is the lowest when compared to Adhesive I (0.104 N/mm) and Adhesive II (0.129 N/mm). However, in terms of mode-II fracture toughness, Adhesive III takes the lead, with a value of *G*_IIC_ = 0.149 N/mm, which surpasses Adhesive I (0.125 N/mm) and nearly matches Adhesive II (0.146 N/mm). Comparing the fracture toughness data of Adhesive III with those of the adhesives reported in Reference [21] and Reference [22], it can be observed that the overall mechanical properties of Adhesive III, particularly its mode-I fracture toughness (*G*_IC_), are lower than those of the adhesives documented in the aforementioned references. Specifically, the mode-I fracture toughness of Adhesive III (*G*_IC_ = 0.0742 N/mm) is not only lower than the corresponding value of the adhesive in Reference [21] but also significantly lower than that of the adhesive in Reference [22]. This reflects that Adhesive III exhibits a certain gap in basic fracture resistance under room temperature conditions.

However, Adhesive III demonstrates a unique advantage in terms of mode-II fracture toughness (*G*_IIC_ = 0.149 N/mm), with its value being significantly higher than the 0.079 N/mm of the adhesive in Reference [22]. This performance difference is attributed to the introduction of specific modified components in the formulation of Adhesive III in this study, which has led to its relatively lower mechanical properties at room temperature.

All the tests were conducted with 5 replicates, and the coefficient of variation (CV) for each set of data is less than 10%, which ensures the consistency and reliability of the results. This performance profile indicates that Adhesive III is well-suited for applications involving shear-dominated load conditions (such as structural joints under sliding stress), even though it has relatively weaker resistance to opening-mode cracks.

## 5. Discussion

**Figure 16:**
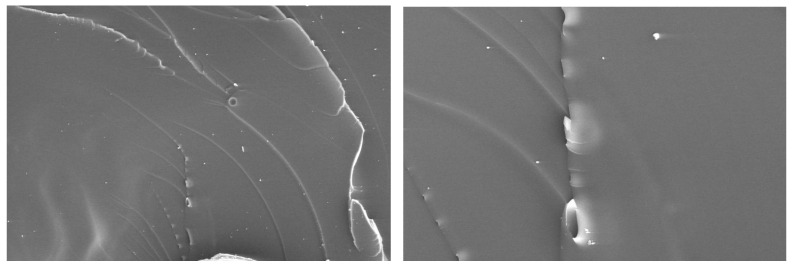
Microscopic morphology of failure surface in tensile test of Adhesive III.

Adhesive III demonstrates superior mechanical performance in bulk tensile tests, with an elastic modulus of 4.31 GPa and ultimate tensile strength exceeding Adhesive I by 126.5% and Adhesive II by 242.9%. This high stiffness and strength are attributed to its molecular design, incorporating a heterocyclic glycidylamine epoxy resin with organosilicon modification and a self-catalytic amine curing agent, which enhances cross-linking density and molecular chain rigidity. This finding is consistent with existing literature on modified epoxy adhesives: Golewski et al. [13] noted that the elastic modulus of organosilicon-modified epoxy adhesives is 20–30% higher than that of conventional amine-cured epoxy adhesives. This aligns with the phenomenon of a low coefficient of variation (CV < 10%) observed in the bulk tensile tests of this study, confirming an improvement in material homogeneity. Morello et al. [14] further verified that heterocyclic epoxy resins can increase cross-linking density, and their stiffness is 45–55% higher compared to traditional epoxy systems. This is consistent with the result in this study that “the elastic modulus of Adhesive III is 21.4% higher than that of Adhesive I”. Its brittle behavior is confirmed by microstructural analysis, revealing “river-like” patterns and smooth surfaces indicative of limited toughness in Figure 16.

**Figure 17:**
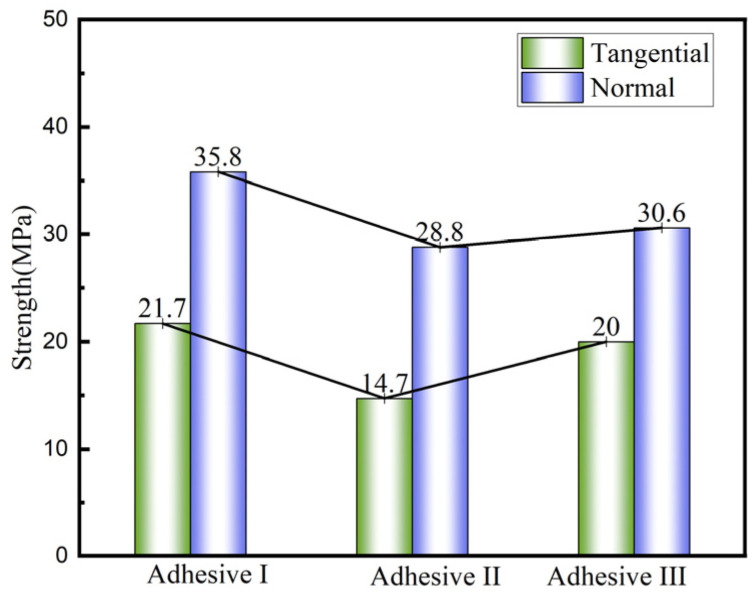
Comparison of the Normal and Tangential Mechanical Properties of Adhesives.

**Figure 18:**
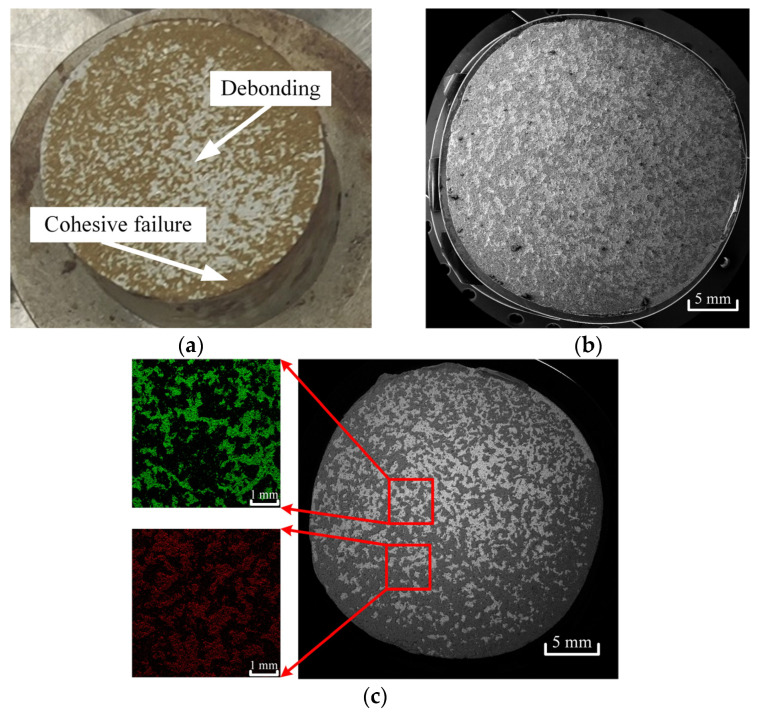
Failure Surfaces of Aluminum Adherend Butt Joints Bonded with Adhesive III Under Normal Tensile Loading: (**a**) Macroscopic cross-sectional morphology; (**b**) Microscopic morphology distribution of the specimen fracture surface under scanning electron microscope, with a magnification of 10×; (**c**) Component distribution map obtained from energy spectrum analysis of the specimen fracture surface, with a magnification of 10×.

**Figure 19:**
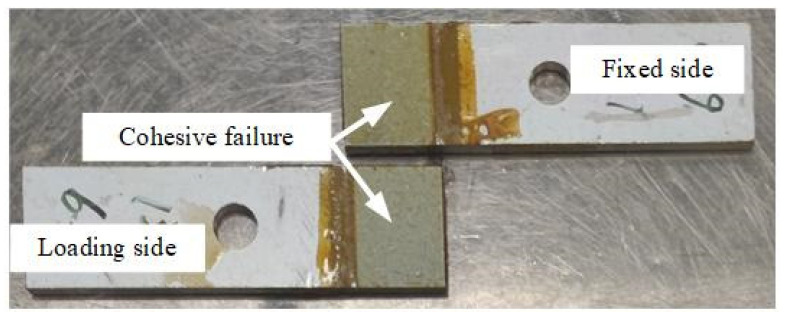
Failure Surfaces of Aluminum Adherend Single Lap Shear Joints Bonded with Adhesive III Under Tangential Shear Loading (Complete Cohesive Failure: Uniform Adhesive Residue on Both Fixed and Loading Side Adherends).

In butt joint tensile and shear tests, Adhesive III’s strength is intermediate (31.3 MPa tensile, 20.0 MPa shear), lower than Adhesive I but higher than Adhesive II. Normal strength consistently exceeds shear strength by 33–107% across all adhesives, attributed to stress concentration at interface edges under shear loading, which accelerates crack initiation, while normal loading distributes stress more uniformly (Figure 17). Fracture surface analysis (Figure 18 and Figure 19) reveals a mixed failure mode under normal loading for Adhesive III, while shear loading results in exclusively cohesive failure, indicating sufficient interfacial bonding strength to withstand shear loads.

To conduct an in-depth study on the mixed failure mode under normal loads, we used a scanning electron microscope (SEM) manufactured by TESCAN (Brno, Czech Republic) with the model MIRA4 to observe the fracture surfaces of specimens exhibiting this mixed failure mode at a magnification of 10× and obtained their microscopic morphologies (Figure 18b). In this figure, the protruding parts are the adhesive that remained attached to the specimens after cohesive failure occurred. Energy dispersive spectroscopy (EDS) analysis was performed using an EDS detector produced by Oxford Instruments (Oxford, UK), and component distribution maps were obtained (Figure 18c). Among them, the green areas represent aluminum element, corresponding to the regions of debonding failure; the red areas represent carbon element, corresponding to the regions of cohesive failure. The component distribution maps derived from the SEM images were subjected to grayscale processing via an image processing program, where cohesive failure areas were converted to black and adhesive failure areas to white. Subsequently, image processing software ImageJ (1.54k) was used to identify the pixel points of the black areas and calculate their area. The results indicate that cohesive failure accounts for 69.32%, while adhesive failure accounts for 30.68%.

Cross-sectional images of Adhesive III were analyzed from specimens subjected to butt joint tensile tests (normal loading, Figure 18) and lap shear tests (tangential shear loading, Figure 19). In accordance with the ASTM D5573 [23] test standard, post-pull-off test specimen images were processed and analyzed. The results showed that 30.68% of the area exhibited debonding, while 69.32% of the area underwent cohesive failure. Under normal loading, the fracture surface in Figure 18 reveals two distinct regions: a dark area, where cohesive failure occurs within the adhesive bulk—evidenced by continuous adhesive residue confirming matrix-dominated cracking—and a light area, indicating adhesive debonding at the interface, characterized by a relatively “clean” surface with minimal residue due to insufficient interfacial bonding. The coexistence of these regions results in a mixed failure mode, with cohesive fracture being predominant, which is consistent with the 69.32% cohesive failure proportion obtained from image analysis. This indicates that, under normal loading conditions, the adhesive bulk, rather than the interface, serves as the more primary load-bearing unit. This failure characteristic is of great significance for evaluating joint durability, as a cohesive-dominated failure mode is generally more predictable than interfacial debonding, with a relatively lower risk of sudden fracture.

In contrast, under tangential shear loading (Figure 19), the substrate surface is uniformly covered by adhesive residue, with no evidence of interfacial debonding. This confirms that failure is exclusively cohesive, occurring within the adhesive bulk rather than at the interface, further highlighting the crucial role of the internal matrix integrity of Adhesive III in ensuring joint strength. Considering the relatively high proportion of cohesive failure, in subsequent work, the overall reliability of the joint could be enhanced by optimizing the bulk toughness of the adhesive (such as adding elastomeric modifiers).

Adhesive III’s lower mode-I fracture toughness (*G*_IC_ = 0.0742 N/mm) suggests limited resistance to crack initiation in opening-mode loading, potentially leading to rapid crack propagation under high peeling loads. Conversely, its superior mode-II fracture toughness (*G*_IIC_ = 0.149 N/mm) indicates enhanced resistance to sliding-mode fracture, likely due to moderate plastic deformation absorbing energy. Compared to composite-metal joints reported in references [11,12,13], Adhesive III excels in strength and mode-II toughness, making it suitable for shear-dominated structures in aerospace thermal protection systems.

## 6. Conclusions

To address the demand for adhesives in the aerospace field, this study systematically evaluated the mechanical properties and adhesive interface failure modes of a novel high-strength epoxy adhesive (DQ622JD-136, hereinafter referred to as Adhesive III) through standardized mechanical tests, with comparisons to conventional adhesives HYJ-16 (Adhesive I) and HYJ-29 (Adhesive II). The core contributions and key findings of the study are summarized as follows:

- The initial null hypothesis H_0_ (stating no significant differences in elastic modulus, bulk ultimate tensile strength, and mode-I/mode-II fracture toughness between Adhesive III and conventional adhesives) was partially rejected through experimental verification, with the experimental results showing both consistencies and inconsistencies with expectations. In terms of consistency with expectations, Adhesive III demonstrated significant advantages in stiffness and tensile strength: its elastic modulus reached 4.31 GPa, which was 21.4% and 55.0% higher than that of Adhesive I and Adhesive II, respectively; its bulk ultimate tensile strength was even 126.5% and 242.9% higher than that of the two conventional adhesives. These results fully confirm that Adhesive III possesses excellent deformation resistance and load-bearing potential, aligning with the initial design expectations.
- However, from the perspective of inconsistencies with expectations and performance trade-offs, Adhesive III has obvious shortcomings. On the one hand, its fracture toughness shows an unbalanced performance: the mode-I fracture toughness is only 0.0742 N/mm, significantly lower than that of Adhesive I (0.104 N/mm) and Adhesive II (0.129 N/mm), indicating weak resistance to the initiation of opening-mode cracks; the mode-II fracture toughness (0.149 N/mm) is only superior to that of Adhesive I (0.125 N/mm) and shows no significant difference from Adhesive II (0.146 N/mm). Overall, it exhibits the unanticipated performance characteristic of “high stiffness—low toughness”. On the other hand, Adhesive III has no obvious yield point and exhibits brittle fracture characteristics during fracture, with “river-like” patterns visible on the fracture surface. This further confirms its insufficient toughness and points out the direction for targeted optimization in subsequent work.
- In addition, results from fracture analysis and joint tests indicate that the failure mode of Adhesive III shows regular characteristics with changes in load type: under normal loading, it exhibits a mixed failure mode of “cohesive failure + interfacial debonding”. Based on scanning electron microscopy (SEM), energy-dispersive spectroscopy (EDS), and image analysis, the proportion of cohesive failure was found to be 69.32%, while that of adhesive failure was 30.68%. Under shear loading, it presents complete cohesive failure. This regularity not only verifies the hypothesis that “the interior of the adhesive layer tends to become a stress weak zone under shear loading” but also provides key experimental basis for the adhesive design of multi-layer structures in aerospace applications. Comprehensively, Adhesive III, with its stable cohesive failure characteristic under shear loading, is suitable for aerospace thermal protection systems dominated by shear loading. However, its relatively low mode-I fracture toughness still requires optimization and improvement in subsequent research.

Adhesive III possesses significant advantages: an elastic modulus of 4.31 GPa, a compressive shear strength of 30.9 MPa, and a 100% cohesive failure characteristic under shear loading. These properties position it as a potential preferred material for multi-layer structures in aerospace thermal protection systems, particularly in shear-dominated regions. In such areas, its high rigidity and shear resistance can compensate for the limitations of traditional medium–low-temperature curing adhesives. However, the limitation of its mode-I fracture toughness cannot be overlooked. Its fracture toughness value (*G*_IC_ = 0.0742 N/mm) is 28.7% lower than that of Adhesive I and 42.5% lower than that of Adhesive II. This shortcoming restricts its application in TPS regions subjected to peeling loads, such as leading-edge components exposed to aerodynamic impact or seams affected by thermal expansion mismatch. In these scenarios, the adhesive may face the risk of rapid crack propagation, which in turn endangers structural integrity.

Adhesive III’s high stiffness, strength, and excellent mode-II fracture toughness make it highly suitable for multilayer structures in aerospace thermal protection systems, where shear and compressive loads predominate. However, its lower mode-I toughness suggests additional design considerations for regions subjected to high peeling or tensile separation forces. This study was conducted at room temperature, excluding the effects of high-temperature aging, humidity, or salt mist corrosion. Long-term performance under cyclic loading was not evaluated. Future research should include high-temperature and cyclic loading tests to validate Adhesive III’s suitability in extreme environments and explore modifications to enhance its mode-I fracture toughness.

Future studies will focus on verifying and optimizing the performance of Adhesive III in extreme aerospace environments, with key priorities including high-temperature mechanical property characterization, thermomechanical coupling tests, and environmental durability studies. Specifically, high-temperature mechanical property characterization will involve testing temperature-dependent changes in elastic modulus, strength, and fracture toughness at typical service temperatures (150–300 °C), with particular attention to the evolution of mode-II toughness under elevated temperatures. Thermomechanical coupling tests will evaluate fatigue resistance and interfacial debonding behavior under dynamic thermal–mechanical interactions via temperature cycling combined with periodic loading while also analyzing the impact of thermal expansion mismatch between the adhesive and substrates. Additionally, environmental durability studies will assess performance degradation after long-term exposure to high temperatures, humidity, or salt mist aging to reveal mechanisms such as oxidation and crosslinking changes, providing critical data to support service life prediction. These efforts will comprehensively verify Adhesive III’s suitability for extreme environments and lay a foundation for its engineering application and performance improvement.

## Figures and Tables

**Table 1:** Manufacturing Process Parameters and Basic Physical Properties of Three Types of Adhesives.

Name	Adhesive I	Adhesive II	Adhesive III
Manufacturer	Aerospace Research Institute of Materials & Processing Technology	Aerospace Research Institute of Materials & Processing Technology	Aerospace Research Institute of Materials & Processing Technology
Mix Ratio/Components	Component A:Component B = 145:13	Component A:Component B = 147:8	Component A:Component B:Component C = 100:29:10
$\mathrm{Density}\;({g/\mathrm{cm}}^{3}$)	1.235	1.044	1.187
Viscosity (mPa·s)	15,868	86,382	8000–15,000
Color	Translucent white	Pale yellow	Pale yellow
Curing Temperature (°C)	23–25	70–75	80–110
Curing Time	Not less than 7 days	Not less than 3 h	23–25 °C for 24 h, followed by stepped curing for not less than 12 h

**Table 2:** Normal Bonding Strength and Fracture Energy of Adhesives.

	Adhesive I	Adhesive II	Adhesive III	Adhesive Used in Reference [22]
$G_{\mathrm{IC}}$ (N/mm)	0.104	0.129	0.0742	0.410
$\mathrm{CV}\;\mathrm{of}\;G_{\mathrm{IC}}$ (%)	6.9	7.8	9.1	0.422
$G_{\mathrm{IIC}}$ (N/mm)	0.125	0.146	0.149	0.079
$\mathrm{CV}\;\mathrm{of}\;G_{\mathrm{IIC}}$ (%)	3.1	1.6	8.3	0.002

## Data Availability

The original contributions presented in this study are included in the article. Further inquiries can be directed to the corresponding author.

## Abbreviations

TPS	Thermal Protection System
TDCB	Tapered Double Cantilever Beam
ENF	End-Notched Flexure
CV	Coefficient of Variation
